# Illuminating spatial A-to-I RNA editing signatures within the *Drosophila* brain

**DOI:** 10.1073/pnas.1811768116

**Published:** 2019-01-18

**Authors:** Anne L. Sapiro, Anat Shmueli, Gilbert Lee Henry, Qin Li, Tali Shalit, Orly Yaron, Yoav Paas, Jin Billy Li, Galit Shohat-Ophir

**Affiliations:** ^a^Department of Genetics, Stanford University, Stanford, CA 94305;; ^b^The Mina and Everard Goodman Faculty of Life Sciences, Bar-Ilan University, 5290002 Ramat-Gan, Israel;; ^c^Janelia Research Campus, Howard Hughes Medical Institute, Ashburn, VA 20147;; ^d^Israel National Center for Personalized Medicine, Weizmann Institute of Science, 7610001 Rehovot, Israel;; ^e^The Institute of Nanotechnology and Advanced Materials, Bar-Ilan University, 5290002 Ramat Gan, Israel;; ^f^The Leslie and Susan Gonda Multidisciplinary Brain Research Center, Bar-Ilan University, 5290002 Ramat-Gan, Israel

**Keywords:** RNA editing, *Drosophila*, neurons

## Abstract

Adenosine-to-inosine (A-to-I) RNA editing, catalyzed by ADAR enzymes, is a ubiquitous mechanism that generates transcriptomic diversity. This process is particularly important for proper neuronal function; however, little is known about how RNA editing is dynamically regulated between the many functionally distinct neuronal populations of the brain. Here, we present a spatial RNA editing map in the *Drosophila* brain and show that different neuronal populations possess distinct RNA editing signatures. After purifying and sequencing RNA from genetically marked groups of neuronal nuclei, we identified a large number of editing sites and compared editing levels in hundreds of transcripts across nine functionally different neuronal populations. We found distinct editing repertoires for each population, including sites in repeat regions of the transcriptome and differential editing in highly conserved and likely functional regions of transcripts that encode essential neuronal genes. These changes are site-specific and not driven by changes in *Adar* expression, suggesting a complex, targeted regulation of editing levels in key transcripts. This fine-tuning of the transcriptome between different neurons by RNA editing may account for functional differences between distinct populations in the brain.

## Significance

A fundamental question in contemporary neuroscience is how the remarkable cellular diversity required for the intricate function of the nervous system is achieved. Here, we bridge the gap between a cellular machinery that is known to diversify the transcriptome and the existence of distinct neuronal populations that compose the *Drosophila* brain. Adenosine-to-inosine (A-to-I) RNA editing is a ubiquitous mechanism that generates transcriptomic diversity in cells by recoding certain adenosines within the pre-mRNA sequence into inosines. We present a spatial map of RNA editing across different neuronal populations in *Drosophila* brain. Each neuronal population has a distinct editing signature, with the majority of differential editing occurring in highly conserved regions of transcripts that encode ion channels and other essential neuronal genes.

The complexity and function of the nervous system is due in part to the existence of various types of neuronal cells with distinct functions, anatomical locations, structures, physiologies, and connectivity. This diversity is accomplished by molecular programs that shape the repertoire of RNA molecules and proteins within each cell, giving rise to populations with distinct molecular signatures. Numerous mechanisms contribute to the genomic, transcriptomic, and proteomic diversity between neuronal populations, including activation of transposable elements, alternative splicing, and RNA modifications (1–3). One particular modification critical to brain function is adenosine-to-inosine (A-to-I) RNA editing, catalyzed by proteins called adenosine deaminases that act on RNA (ADARs), which are conserved across metazoans (4, 5). The resulting inosines are read by the cellular machinery as guanosines, leading to a variety of consequences, including altered splicing and gene expression and changes to the amino acid sequences of proteins (6, 7).

Thousands of RNA editing sites have been discovered in *Drosophila* (8–15), and the loss of ADAR editing results in mainly neuronal and behavioral phenotypes (5, 16). Many of these sites are predicted to cause nonsynonymous protein-coding (“recoding”) changes in genes that are expressed and function primarily in neurons, such as ion channels and presynaptic proteins involved in neurotransmission. Evolutionary analysis of editing across multiple *Drosophila* species indicates that many of the recoding events in neuronal genes are being selected for over evolution, suggesting that their editing may be functionally important (12–14).

Studies indicate that editing modulates the kinetics of the voltage-dependent potassium channels Shaker and Shab (17, 18); the agonist potency of the GABA-gated chloride channel, Rdl (19); and the voltage sensitivity and closing kinetics of the sodium channel Paralytic (Para) (20). While there are more protein-recoding editing events in flies than in mammals, a number of mammalian ion channels also undergo functionally important RNA editing events, which can be dynamically regulated across brain tissues (21, 22); yet, the regulation of a particular editing site may not be fully assessed at the entire tissue level. Editing levels are known to differ between neurons and glial cells (23), but little is known about the diversity and functional importance of this process in different neuronal populations. So far, RNA editing profiling of *Drosophila* neurons has faced the technical difficulty of reliably defining and isolating certain neuronal populations out of many in sufficient quantity, and thus editing level measurements typically represent an average of editing from large brain regions or whole brain tissue.

Here, we utilized a battery of *Gal4* drivers and refined the INTACT (Isolation of Nuclei Tagged in A specific Cell Type) method (24) to analyze the spatial distribution of editing events among nine different neuronal populations taken from adult fly brains. To examine the editing levels of thousands of known and novel editing sites, we deployed two complementary approaches: RNA-sequencing (RNA-seq) to quantify editing levels in highly expressed transcripts across the different neuronal populations and microfluidic multiplex PCR and sequencing (mmPCR-seq) to gain highly accurate editing level measurements at targeted sites (25). We identified editing sites using the RNA-seq data and then determined editing levels at these sites and previously identified sites through either mmPCR-seq or RNA-seq. We found that each neuronal population has a unique RNA editing signature composed of distinct editing levels of specific sites in neuronal transcripts, some of which harbor unique combinations of multiple editing sites. Many of these regulated sites have been predicted to be functional. We found evidence for coregulation of nearby sites in the same transcripts and identified instances where different subunits of a certain neuronal machinery are edited differentially in distinct population of neurons. Furthermore, we show that these editing level differences are unlikely to be caused by changes in *Adar* expression, suggesting other factors, including differentially expressed RNA binding proteins, may regulate ADAR to fine-tune editing levels across different populations of neurons.

## Results

### Isolation of RNA from Discrete Nuclei-Tagged Neuronal Populations.

**Fig. 1.**
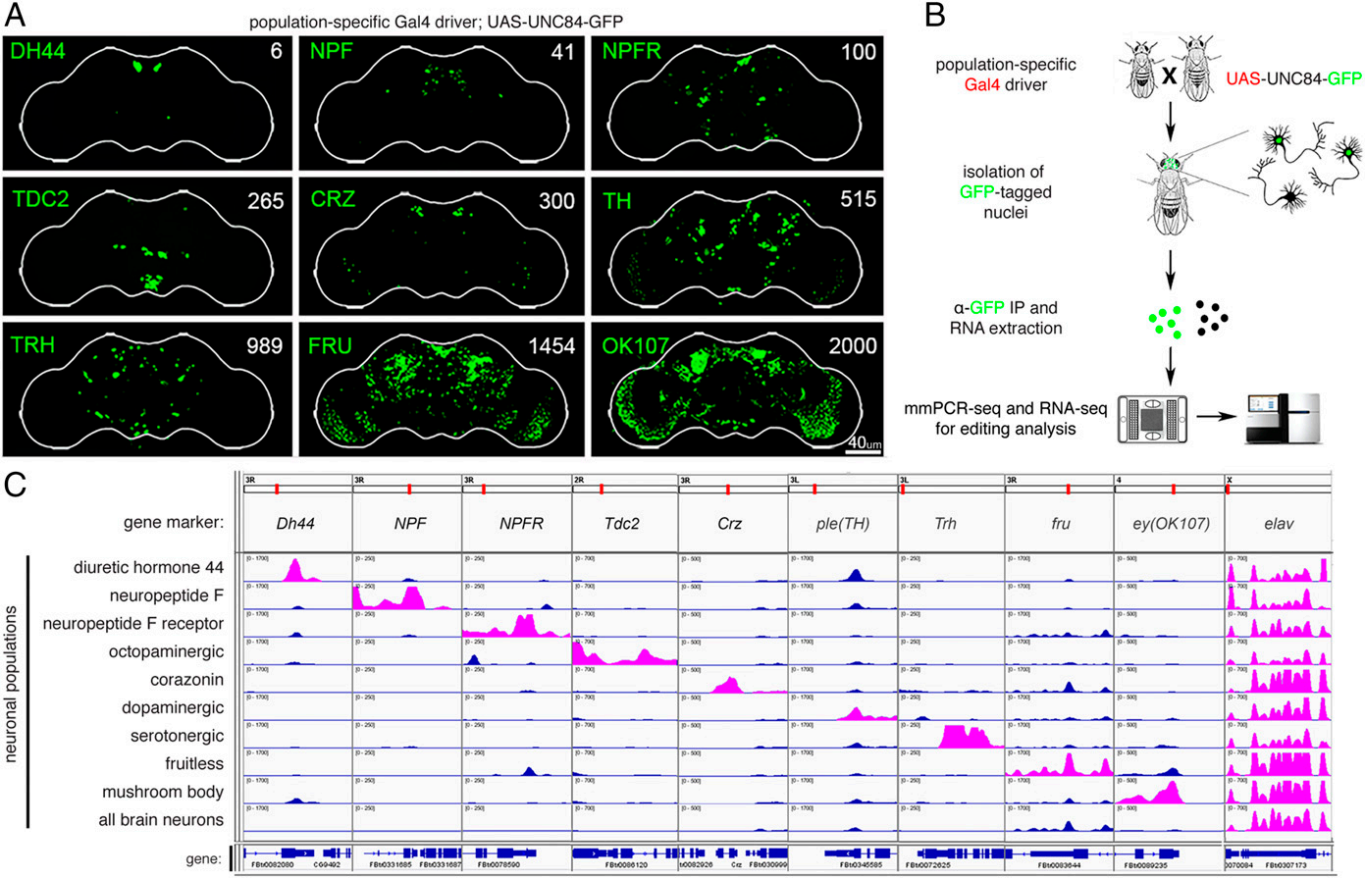
Isolation of RNA from distinct neuronal populations. (*A*) Confocal images of GFP-marked nuclei in fly brains from the nine neuronal populations used in this study. *Gal4* drivers are listed on the left of each image, with the number of cells in each neuronal population listed on the right. (Scale bar: 40 µm.) (*B*) Schematic of workflow for isolating RNA from discrete neuronal populations and RNA editing analysis. (*C*) Visualization of RNA-seq reads from the nine cell populations and Elav control at marker genes for the 10 groups. Reads of the relevant marker genes for each population listed on the left are shown in pink.

We and others had measured editing levels in whole fly heads and brains (10, 26), but treating the brain as one unit prevented us from pinpointing editing sites that are differentially regulated between distinct populations of neurons, which may reflect their functional importance. To reveal RNA editing level variation between types of neurons, we used *Gal4* drivers to mark and isolate different subsets of neurons within the fly brain. The chosen neuronal populations regulate various aspects of behavior and physiology and are composed of varying numbers of cells with distinct anatomy and connectivity across the brain (Fig. 1*A*). The largest population we studied are the mushroom body neurons (marked by *OK107-Gal4*) (27), which serve as the integration center for many behaviors and have a major role in learning and memory (28), and the Fruitless (*fru-Gal4*) neurons, which are implicated in specifying sexual behavior in male and female flies and comprise ∼2% of central nervous system neurons. We chose four populations of neurons associated with neuropeptide signaling, Neuropeptide F (*NPF-Gal4*), Neuropeptide F Receptor (*NPFR-Gal4*), Diuretic hormone 44 (*Dh44-Gal4*), and Corazonin neuropeptide (*Crz-Gal4*), which regulate different aspects of motivational behaviors and stress response and represent only a small number of neurons in the brain (29). We also chose three populations expressing neurotransmitters, dopamine (*TH-Gal4*), serotonin (*Trh-Gal4*), and octopamine (*Tdc2-Gal4*), which mediate a broad range of innate and learned behaviors as well as regulate homeostatic responses. We also used a pan-neuronal driver (*elav-Gal4*) as a reference for whole brain neurons.

There are several approaches that allow for the isolation of genetically marked subsets of neurons, including manual sorting (30), FACS (31), and ribosome tagging (32). Since RNA editing is a cotranscriptional process that takes place in the nucleus, we chose to analyze newly formed RNA transcripts residing within neuronal nuclei. For that purpose, we used the INTACT method (24). This technique utilizes specific *Gal4* drivers to mark neuronal nuclei with a genetically encoded nuclear tag (UNC84-GFP) that can then be purified by immunoprecipitation. We improved upon the published INTACT protocol by adding a purification step that minimized nonspecific binding of cytoplasmic debris and fragments of broken nuclei (*Methods* and *SI Appendix*, *Extended Methods*). We isolated nuclear RNA from 10 specific neuronal populations and used two complementary methods to measure RNA editing across neuronal populations, RNA-seq and mmPCR-seq (25) (Fig. 1*B*). We used RNA-seq to measure RNA editing in highly expressed transcripts and mmPCR-seq to obtain highly accurate editing level measurements independent of gene expression at 605 loci (33) harboring known editing sites.

To validate our approach in isolating distinct population of neurons, we compared the expression of marker genes across the transcriptome of the 10 different populations of neurons. As expected, the marker genes showed population-specific expression, while the pan-neuronal marker *elav* was evenly expressed in all groups of neurons (Fig. 1*C*). We saw enrichment of the desired markers even for low-abundance populations such as NPF or Dh44 neurons, suggesting that we successfully captured the transcriptomes of these neuronal populations. Some neuronal populations, like the one marked by *TH-Gal4*, had partial overlap with other neuronal populations, as can be seen by the expression of the TH marker gene across several neuronal populations.

### Identification of Editing Sites from Distinct Neuronal Populations.

**Fig. 2.**
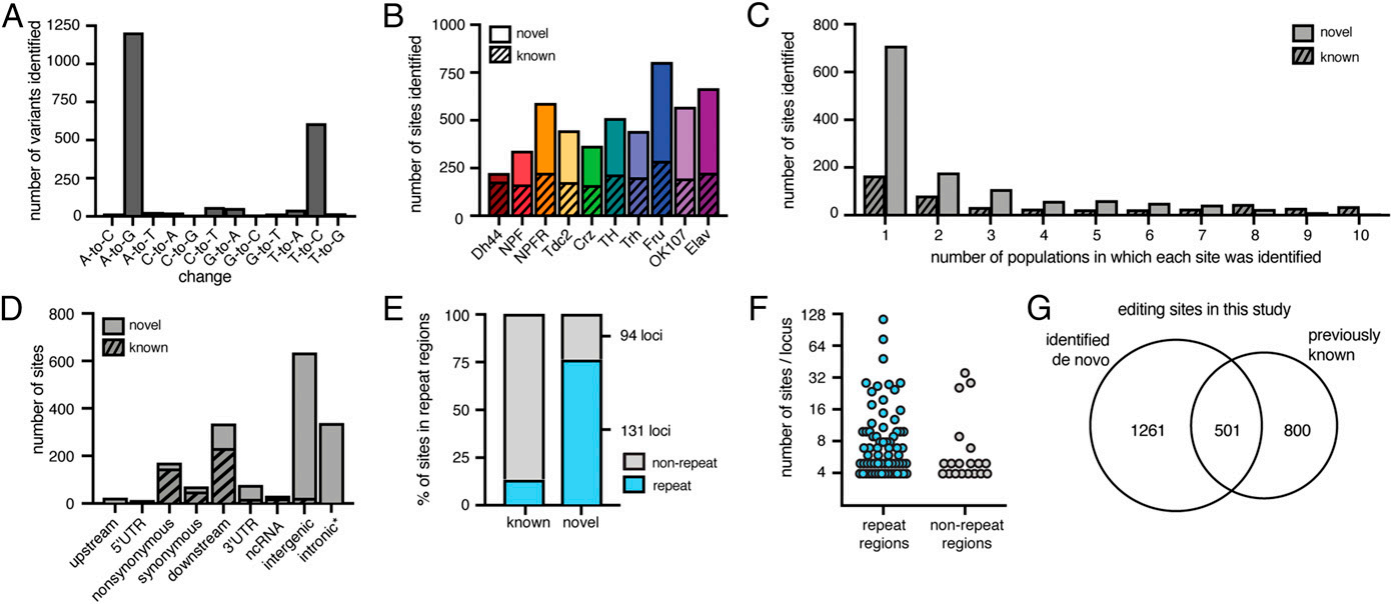
Identification of RNA editing sites from distinct neuronal populations. (*A*) The total number of all variants identified de novo. (*B*) The number of editing sites identified de novo from each population, split into known sites in stripes and novel sites in solids. (*C*) Histogram of the number populations in which each known site and novel site was identified de novo. (*D*) The number of known and novel sites found in each annotated location. *353 sites annotated as intronic are found in the *Myo81F* heterochromatic region of chr3R. (*E*) The percentage of known and novel sites identified by our pipeline that overlap annotated repeat regions (blue) or do not (gray). (*F*) The number of novel editing sites found within each locus that contained at least four sites, for loci overlapping repeat regions and nonrepeat regions. *y* axis is log_2_ scale. (*G*) Venn diagram of editing sites identified de novo and known editing sites used in this study.

We hypothesized that RNAs from distinct neuronal populations would include RNA editing sites that were previously undetected because whole-brain sequencing does not provide adequate coverage of editing sites that are only edited or expressed in a small number of cells. We modified our previously developed computational pipeline to identify editing sites from the transcriptomes of the 10 neuronal groups (*SI Appendix*, *Supplementary Note* and Fig. S1). From all populations combined, we identified 2,058 variants of all possible base conversions, 88% of which were A-to-G or T-to-C and thus indicative of A-to-I editing events (Fig. 2*A*). These sites included both known and novel editing sites in each neuronal population (Dataset S1). We found between 161 (in Crz) and 287 (in Fru) known editing sites and 46 (in Dh44) and 518 (in Fru) novel sites in each neuronal population (Fig. 2*B*). Many of the novel sites were identified in only one neuronal population, whereas known sites were more often identified repeatedly in multiple neuronal populations by our pipeline (Fig. 2*C*), demonstrating that sequencing each distinct neuronal population facilitated the discovery of additional sites. The majority of the novel sites did not overlap annotated regions of the transcriptome (Fig. 2*D*), with 76% overlapping repetitive regions of the genome, compared with 13% of the known sites (Fig. 2*E*). We found that our novel sites grouped into 225 loci (with <100 bases between adjacent editing sites), with repetitive loci often containing large numbers of sites, including one locus having as many as 116 editing sites (Fig. 2*F*). In total, we identified 1,762 editing sites, finding 501 known editing sites and 1,261 novel sites (Fig. 2*G*). Because the de novo identification pipeline included stringent filters, we also measured editing levels at known sites with high coverage in RNA-seq and used mmPCR-seq to measure editing levels at known sites that were not highly covered in RNA-seq. These strategies led us to include an additional 800 known editing sites in our downstream comparative analysis.

### RNA Editing Levels Differ Between Neuronal Populations in the Fly Brain.

**Fig. 3.**
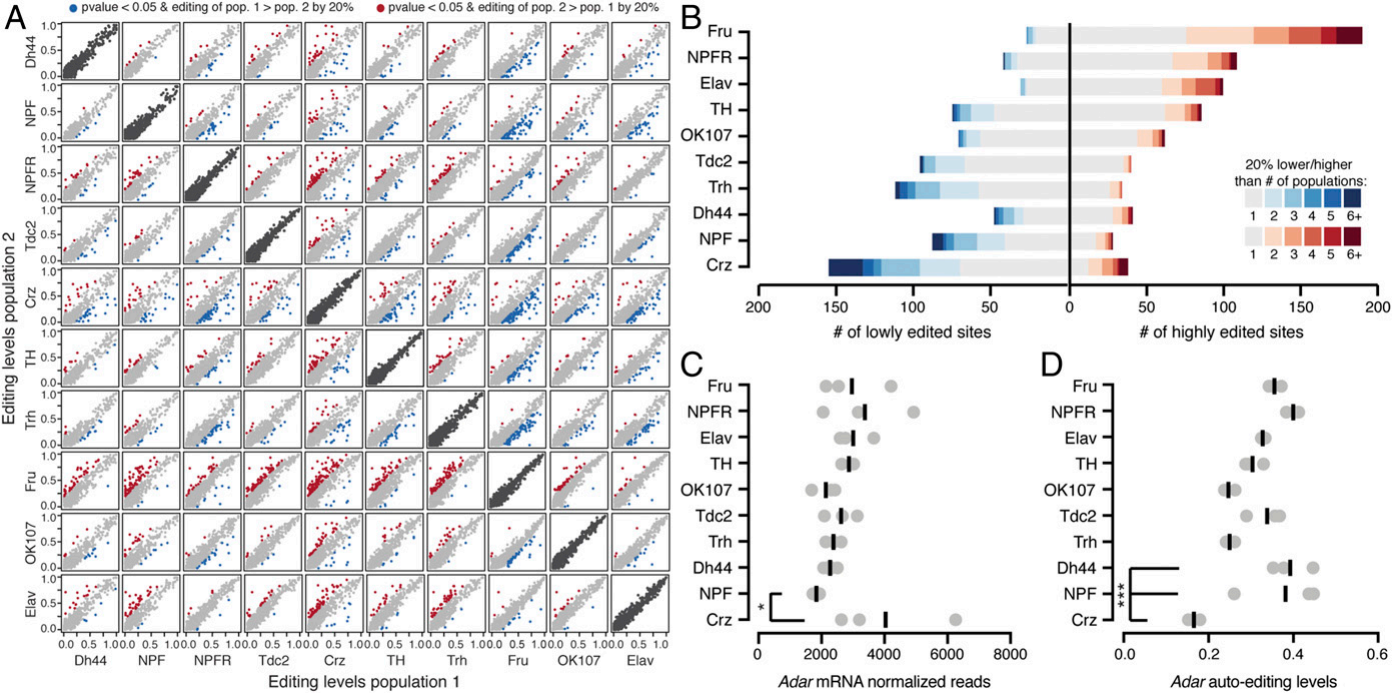
RNA editing level differences between neuronal populations. (*A*) Pairwise comparisons of editing levels from three combined replicates of mmPCR-seq or RNA-seq between 10 populations. Red and blue dots are editing sites that differ by >20% editing between populations with *P* < 0.05 (Fisher’s exact tests), and gray dots are sites with <20% editing change. Dark gray dots are representative biological replicates of each population. (*B*) The number of editing sites that are more highly or lowly edited in each population listed on the left compared with all other populations. Shades of blue and red represent the number of populations in which each site differs in pairwise comparisons. (*C*) *Adar* mRNA normalized read counts from RNA-seq of each population. Each dot is one replicate with bars representing the mean. *P < 0.05 (Wald test). (*D*) Editing levels at the *Adar* auto-editing site at chrX:1781840 in all populations. Each dot is one replicate, with bars representing the mean. ***P < 0.001 (Fisher’s exact test) and editing change >20%.

We calculated RNA editing levels using both RNA-seq and mmPCR-seq by determining the fraction of G reads over the total number of reads at each known and novel site. Both RNA- and mmPCR-seq editing level measurements were highly reproducible between three biological replicates from each neuronal population (*SI Appendix*, Fig. S2). In the subset of editing sites that were covered in both the RNA- and mmPCR-seq, editing levels were highly reproducible between the two methods. To compare editing levels between neuronal populations, we looked at a total of 1,036 editing sites that were covered by either mmPCR- or RNA-seq in at least 7 of 10 different neuronal populations with 20× coverage and editing levels that were reproducible between replicates (Dataset S2). Pairwise comparisons of editing levels between all neuronal populations revealed that 271 editing sites (26% of sites queried) had statistically significant differences of at least 20% in their editing levels between at least two different neuronal populations (Fig. 3*A*). To understand which neuronal populations showed the largest differences, we counted the number of times the same site was differentially edited between each neuronal population in its pairwise comparisons with all other populations. Fig. 3*B* shows the number of sites with decreased or increased editing of at least 20% between each of the 10 neuronal populations and every other population as well as the number of other populations from which each site differed. We found that Fru neurons were the most heavily weighted toward increased editing levels, with 190 editing sites that showed higher editing in Fru than other neuronal populations. Crz neurons were the most skewed toward lower editing than the other neuronal populations, where 155 editing sites had decreased editing compared with at least one other neuronal population. The other neuronal populations had between 88 and 160 editing sites that were differentially edited from at least one other neuronal population (Fig. 3*B*). We also performed principal component analysis on editing levels in all 10 neuronal populations and observed clustering patterns similar to our pairwise editing comparisons (*SI Appendix*, Fig. S3).

To test whether editing level differences between these neuronal populations stem from variation in ADAR levels, we determined *Adar* mRNA expression levels in these populations (Fig. 3*C*). We found that *Adar* levels were similar between neuronal populations, with no correlation between *Adar* expression and the observed differences in overall editing levels between the different neuronal populations (Fig. 3*C* and *SI Appendix*, Fig. S4*A*). Next, we tested whether the differences between the populations could be explained by variation in ADAR enzymatic activity, which is decreased upon auto-editing within its own transcript (16). Like *Adar* expression, auto-editing levels were similar between populations, with Crz neurons having the lowest auto-editing levels (Fig. 3*D* and *SI Appendix*, Fig. S4*B*). This lower auto-editing level was expected to contribute to increased, rather than decreased, editing in Crz; therefore, we concluded that auto-editing levels were not the main cause for the observed differences in editing levels. Furthermore, we found no correlation between the number of cells in each neuronal population and overall editing levels (*SI Appendix*, Fig. S4*C*).

In addition to ADAR, a number of RNA binding proteins are known to regulate editing levels at individual editing sites, and RNA binding proteins in general are good candidates for modulators of editing on a site-by-site basis (34). Comparing the expression levels of RNA binding proteins across neuronal populations, we found 105 different RNA binding proteins that were at least twofold differentially expressed between at least two neuronal populations in pairwise comparisons. These differences included increased expression of *pum*, *orb*, and *Rbp6* in Crz neurons over all other populations (*SI Appendix*, Fig. S5 and Dataset S3). These RNA binding proteins may serve as candidate *trans* regulators of editing levels at the 271 editing sites that were found to be differentially edited between the different neuronal populations.

### Identifying Unique Regulation of Editing Events in Different Neuronal Populations.

**Fig. 4.**
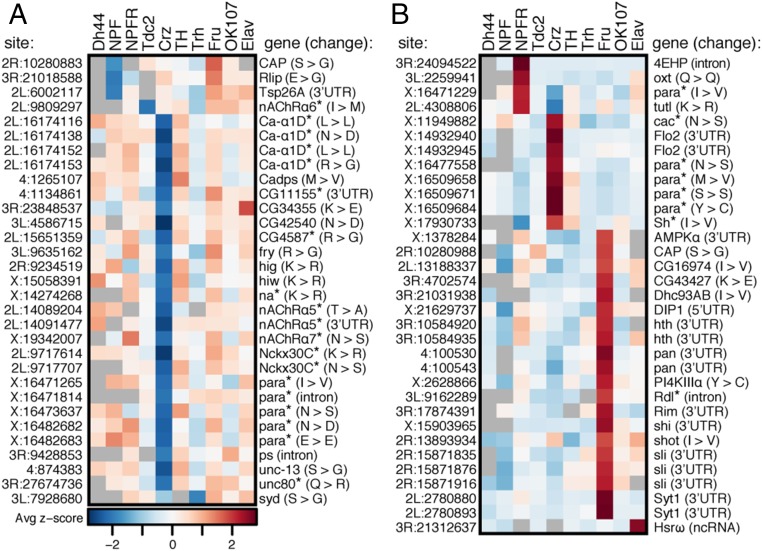
Population-specific editing level differences. Average *z* score of replicate editing levels at sites where one population shows a population-specific decrease in editing (*A*) or a population-specific increase in editing (*B*) is shown. *Genes are involved in ion transport.

We sought to identify sites that were uniquely regulated in one neuronal population compared with all others. We calculated z scores to determine how much each replicate of each population of neurons differed from the mean of all population replicates at each site (Dataset S4). We identified 31 editing sites that were lowly edited in one population (Fig. 4*A*) and 33 sites that were highly edited in one population (Fig. 4*B*). The majority of both the lowly and the highly edited sites were found in Crz and Fru neurons, respectively, consistent with our previous analysis (Fig. 3*B*). The 64 sites with population-specific editing were enriched for nonsynonymous editing events, with 56% of sites predicted to change protein-coding sequences compared with 36% of the total sites we tested (*P* = 0.0010, χ^2^ test). These sites were depleted of intronic sites, with 6% of sites found in introns vs. 28% in the tested population (*P* = 0.0015, χ^2^ test). Synonymous sites, UTR sites, and noncoding RNA sites were neither enriched nor depleted in the set of population-specific sites. To further characterize the list of population-specific editing differences, we performed Gene Ontology (GO) analysis of the genes containing these differentially edited sites. We found that Crz-specific editing sites were located within genes enriched for multiple GO terms related to the regulation of membrane potential and cation transmembrane transport above a background of edited genes in this dataset (Dataset S5). Fru-specific editing sites were found more often in transcripts with roles in cell signaling and differentiation, but they did not show any significant enrichment.

While editing differences between tissues have been associated with expression differences of edited transcripts (35), the majority of transcripts with population-specific editing did not show differential expression in the neuronal population in which they were uniquely edited (*SI Appendix*, Fig. S6 and Dataset S3). However, five transcripts that were edited differently in Crz neurons were also more highly expressed in Crz neurons than in all of the other populations: *CG34355*, *Flo2*, *para*, *nAChRα7*, and *Nckx30C*. These transcripts contained sites with both decreased and increased editing levels, implying that site-specific editing changes cannot be simply explained by the relative abundance of the transcript within a specific neuronal population.

### Coregulation of Clustered Editing Sites.

**Fig. 5.**
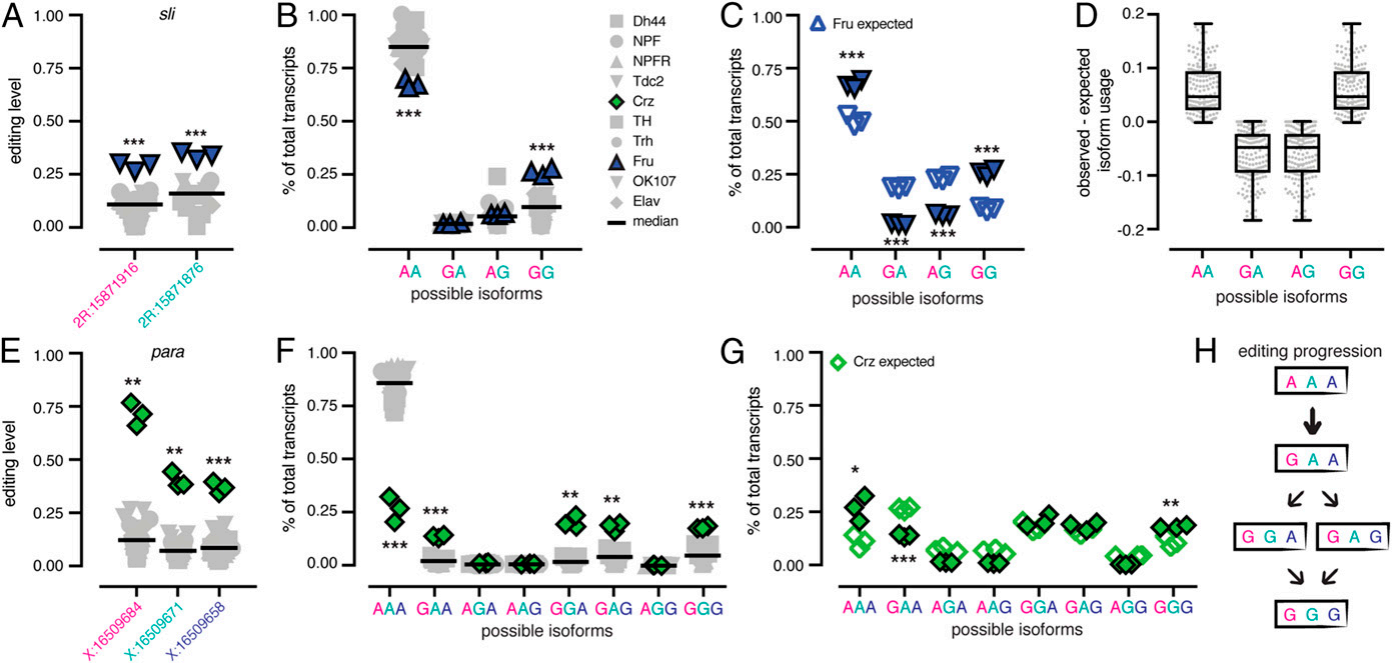
Coregulation of proximal editing sites. (*A*) Editing levels across all replicates of all populations at a cluster of two editing sites in *sli* with Fru in blue. ***P < 0.001 (Welch’s *t* tests). Bars represent median of all replicates. (*B*) The percentage of total transcripts using each possible editing isoform at the two sites in all populations. ***P < 0.001 (Welch’s *t* tests) and mean difference > 10%. (*C*) Observed and expected isoform usage in Fru neurons. ***P < 0.001 (Student’s *t* tests). (*D*) Tukey’s boxplots of the difference between the observed and expected isoform usage for four isoforms in six clusters of coregulated sites in all populations. (*E*) Editing levels across all replicates of all populations at a cluster of three editing sites in *para*, with Crz in green. **P < 0.01; **P < 0.001 (Welch’s *t* test). (*F*) The percentage of total transcripts using each possible editing isoform at the three sites in all populations. **P < 0.01; ***P < 0.001 (Welch’s *t* tests) with mean difference > 10%. (*G*) The observed and expected isoform usage in Crz neurons. *P < 0.05; **P < 0.01; ***P < 0.001 (Student’s *t* tests). (*H*) A model for editing at the cluster of three sites showing editing at the first site is critical for editing at the other sites in the cluster.

Of the 64 sites that showed population-specific editing levels, we found a total of 19 sites in eight different groups that were located within 40 bases of at least one other population-specific site. Since nearby editing events often occur within the same physical transcript (36), we examined whether these groups of sites showed similar population-specific editing trends due to coregulation of the editing events in the clusters. We measured the usage of each possible editing isoform in all neuronal populations for clusters of sites that could be found within the same mmPCR-seq amplicon (Dataset S6). First, we looked at a cluster of two sites in the *sli* transcript that are 40 bases apart and were both highly edited in Fru neurons. These sites were edited at 29% and 34% in Fru, whereas the median editing levels across all populations were 10% and 18%, respectively (Fig. 5*A*). We calculated the usage of the four possible editing isoforms covering these two sites, and we found that in Fru, the AA isoform was found less often than in the other populations (67% compared with the median 84%) and the GG isoform was found more often than in the other populations (26% compared with the median 10%) (Fig. 5*B*). Based on the editing levels measured at the two sites independently, we would expect the AA and GG isoforms to represent 50% and 8% of the total number of transcripts; instead, we found both the AA and GG to be overrepresented by 17%, with a concomitant decrease in AG and GA transcripts (Fig. 5*C*), suggesting that the editing events at the two sites are in fact linked. We then measured the differences in the observed vs. the expected percentages of isoform usage in the five other clusters of two sites that appeared to be coregulated for all populations. We found that, in all populations, these sites also showed an overrepresentation of AA and GG isoforms and an underrepresentation of AG and GA isoforms (Fig. 5*D* and *SI Appendix*, Fig. S7 *A*–*E*), confirming that in these clusters, ADAR preferentially edits either both sites together or neither site.

In addition to the coregulated clusters of two sites, we also identified two larger clusters of sites that showed similar evidence of coregulation, including a cluster of four sites in *Ca-α1D* (*SI Appendix*, Fig. S7*F*) and a cluster of three sites in *para*. This three-site cluster showed editing increases of 58%, 32%, and 27% over the median editing levels of the other neuronal populations (Fig. 5*E*). We found that, at the isoform level, these editing increases at the three sites led to a 59% decrease in completely unedited transcripts (AAA) from the median level of all populations and a similar increase (between 12% and 19%) of four different editing isoforms: GAA, GGA, GAG, and GGG (Fig. 5*F*). Similar to the clusters with two sites, we found that the completely unedited isoform and the completely edited isoform (AAA and GGG) were overrepresented, while isoforms with only one editing event (GAA, AGA, and AAG) were underrepresented (Fig. 5*G*). From this data, we can postulate a progression of editing at these three sites. Since all edited isoforms included editing at the first site, we propose that this site is edited first and required for editing at one or both of the second and third sites (Fig. 5*H*).

### Differential RNA Editing in Transcripts Involved in Neuronal Transmission.

**Fig. 6.**
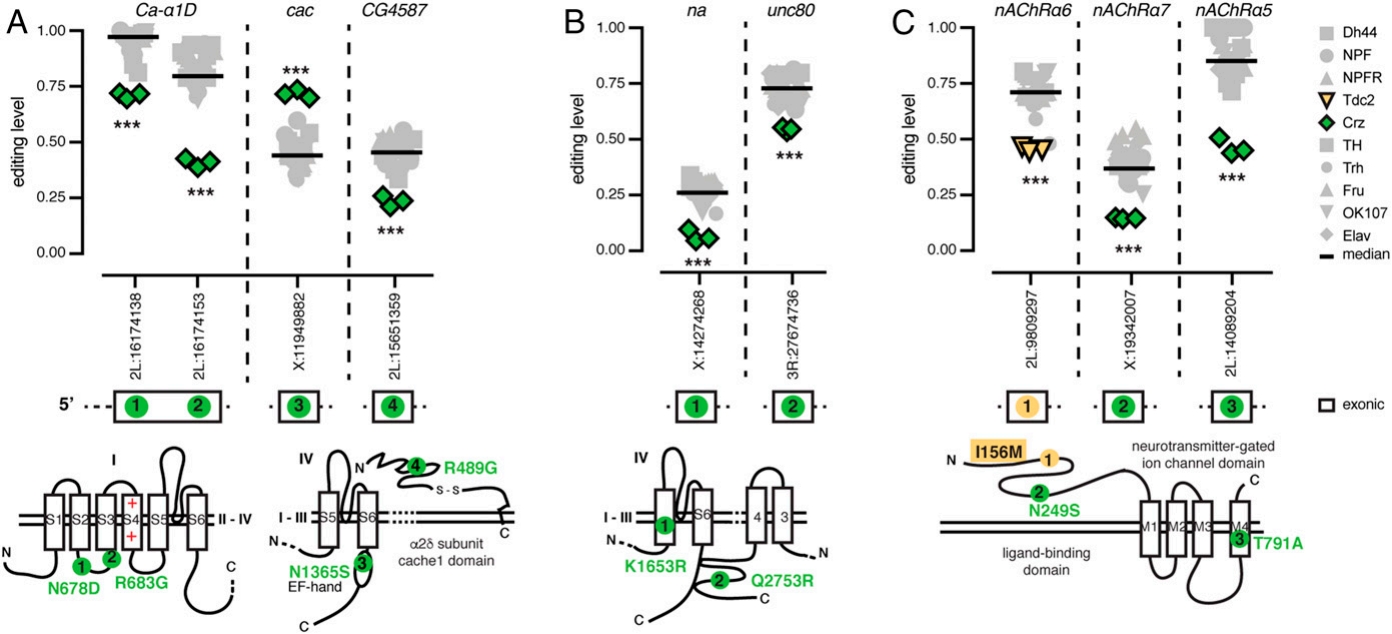
Coregulation of RNA editing in related proteins. (*A*) RNA editing levels at sites within transcripts encoding calcium-gated ion-channel subunits, Ca-α1D, cac, and CG4587, with Crz in green. (*B*) RNA editing levels at sites within transcripts encoding sodium leak channel components, na and Unc80. (*C*) RNA editing levels at sites within transcripts encoding nAChR subunits, nAChRα6 (Tdc2 in yellow), nAChRα5, and nAChRα7. Bars are median editing of all populations. ***P < 0.001 (Welch’s *t* test). Location of amino acids affected by editing as determined by Uniprot (*SI Appendix*, Table S1) are marked on protein drawings, as numbered in transcripts above.

A substantial proportion of the transcripts with population-specific editing have roles in neuronal transmission, so we wanted to explore the consequences of these editing differences. We compared our population-specific editing sites to three recent studies that used computational strategies to predict editing events in *Drosophila* that are likely to be functional because they are found in conserved regions of the genome, are conserved as editing events throughout multiple *Drosophila* species, or are in regions under positive selection (12–14). Of the 64 population-specific editing sites, 41 sites were predicted to be likely functional by at least one of these studies, while only 8 sites showed evidence against functionality (the remaining 15 were not studied; Dataset S2). Of the sites that were predicted to be functional, many are found in transcripts that encode proteins that are critical for neuronal function, and some are known to function together within the same multiprotein complex or in the same pathway. In Crz neurons, *Ca-α1D*, which encodes an α subunit of a voltage-gated calcium channel, had two editing sites that showed a decrease in editing of 26% and 42% from median editing levels across all neuronal populations (Fig. 6*A*). We also observed an editing increase of 27% at a site within the EF-hand calcium-sensing domain of the voltage-gated calcium channel, cacophony (cac). Furthermore, we observed a decrease in editing of 22% from the median level of all populations in *CG4587*, which encodes an auxiliary α2δ subunit of voltage-gated calcium channels (Fig. 6*A*). These editing events have the potential to alter calcium ion flow within these neurons. The population-specific editing of the different subunits suggests that each neuronal population possesses a unique mixture of voltage-gated calcium channel isoforms. We also observed a similar regulation of editing at putatively functional sites in two transcripts encoding the sodium leak channel complex. One editing site within each of the narrow abdomen (na) α subunit and its auxiliary subunit Unc80 (37) showed decreased editing of 19% in Crz neurons from the median levels of all populations (Fig. 6*B*). Various acetylcholine-gated ion channels (nicotinic acetylcholine receptors; nAChRs) also showed editing differences between populations. *nAChRα6* and *nAChRα7* showed a decrease in editing in Tdc2 of 27% and in Crz of 21%, respectively, at sites that change amino acids in the ligand-binding domains of these related proteins, while the *nAChRα5* showed 40% lower editing in Crz at one site predicted to change an amino acid in the ion-channel pore domain (Fig. 6*C*). The identification of differential editing of several protein subunits that function together highlights the strength and complexity of RNA editing in diversifying the proteomic architecture. The composition of neuronal machineries within a certain neuronal population is determined by the distribution of edited vs. nonedited forms of multiple subunits associating within the same multiprotein complex. These data may indicate coregulation of editing across related transcripts in different neuronal populations.

### Editing Differences Suggest Functional Differences.

**Fig. 7.**
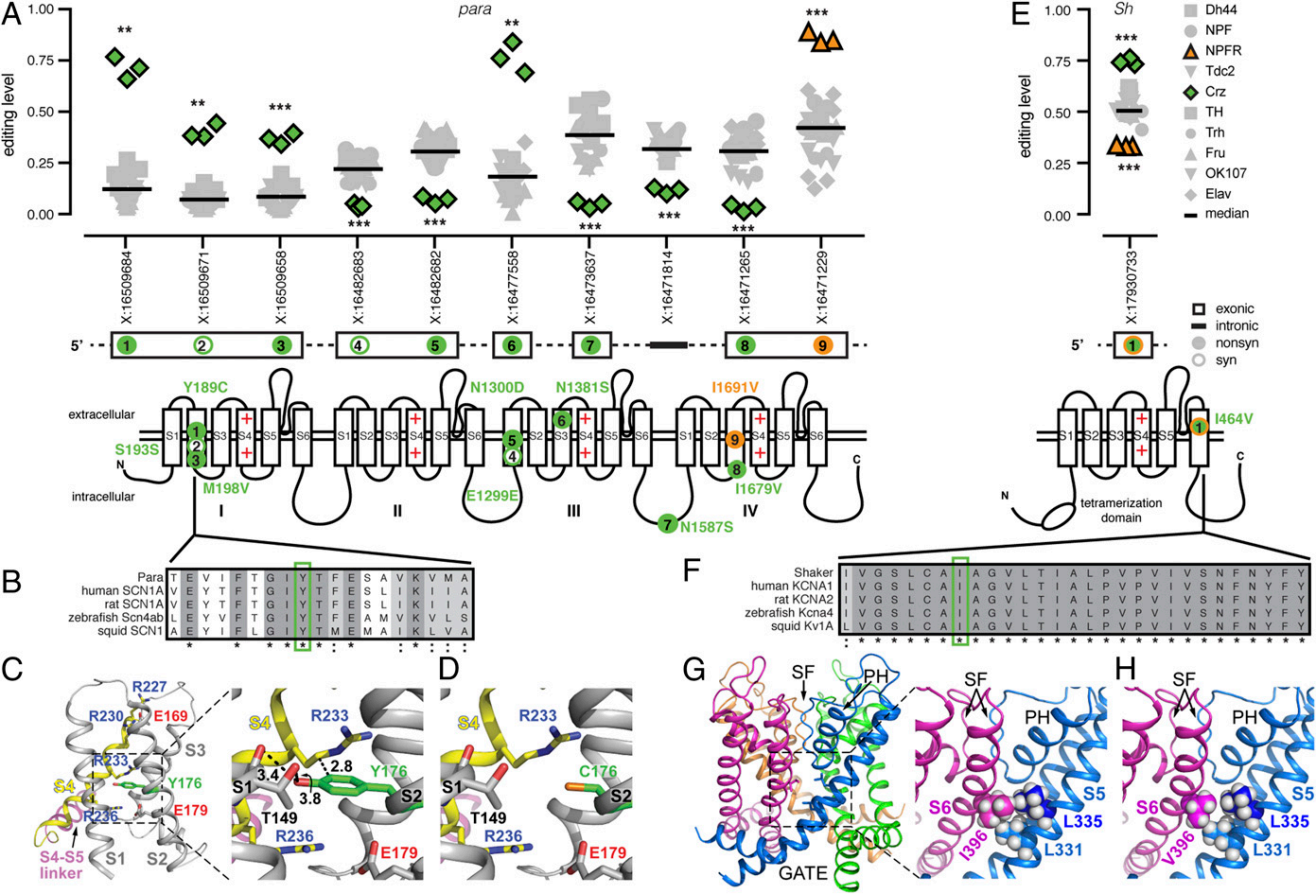
RNA editing in voltage-gated ion channels Para and Sh. (*A*) RNA editing levels at population-specific editing sites in *paralytic*. Crz is in green, and NPFR is in orange for significant sites. Bars are median editing levels of all populations. **P < 0.01; ***P < 0.001 (Welch’s *t* tests). Location of amino acids affected by editing are marked on protein drawings, as numbered in transcripts above. (*B*) Amino acid conservation within the S2 transmembrane domain of repeat I across voltage-gated sodium channels of five species with Tyr^189^ highlighted in green. (*C*) Ribbon diagram of the VSD (side view of S1–S4) of Para mapped onto the 3D structure of the homologous voltage-gated Na^+^ channel from *Periplaneta Americana*. Tyr^176^ (green) is homologous to *D. melanogaster* Tyr^189^. (*C*, *Right*) Magnification showing the potential interactions (dashed lines) of Tyr^176^ (in S2) with Thr^149^ (in S1) and Arg^233^ (in S4). (*D*) Same as in *C*, but reflecting the RNA editing of Tyr^176^ to Cys. Carbon atoms are gray or yellow. Oxygen, nitrogen, and sulfur atoms are in red, blue, and orange, respectively. Hydrogen atoms were removed for clarity. Numbers near dashed lines show distances in angstroms. (*E*) RNA editing levels at an editing site with population-specific editing in *Shaker*. (*F*) Amino acid conservation within the S6 transmembrane domain across voltage-gated potassium channels of five species with Ile^464^ highlighted in green. (*G*) Ribbon diagram of the pore domain of Shaker mapped onto the 3D structure of the *R. norvegicus* Kv1.2 voltage-gated K^+^ channel shown from the side, with the four identical subunits colored differently. PH, pore helix; SF, selectivity filter. (*G*, *Right*) Magnification showing Ile^396^ (homologous to Ile^464^) of the S6 segment and its potential van der Waals interactions with Leu^331^ and Leu^335^ of S5 in the adjacent subunit. (*H*) Same as in *G*, but reflecting the RNA editing of Ile^396^ to Val.

Numerous editing sites with differential editing levels between neuronal populations occurred within voltage-gated ion channels such as Para and Shaker. Voltage-gated ion channels are composed of four subunits or four linked subunit-like repeats, each of which contains six transmembrane segments (S1–S6) (38). In Crz neurons, the transcript encoding the voltage-gated sodium channel Para was differentially edited at 10 different sites along its transcript, with 6 sites showing decreased editing and 4 sites showing increased editing levels. The first site, changing Tyr^189^ to Cys, showed a remarkable 58% editing increase in Crz neurons over the median level in other neuronal populations, while the last site, an Ile^1691^ to Val change in the S3 of repeat IV, showed a 45% increase in NPFR neurons (Fig. 7*A*). The Tyr^189^ in S2 of the voltage-sensor domain (VSD) of repeat I is highly conserved across different species (Fig. 7*B*), leading us to hypothesize that an editing change might alter protein function.

To predict the functional consequences of recoding Tyr^189^ into a cysteine residue (Y189C), we mapped the edited amino acid onto the 3D structure of the homologous voltage-gated sodium channel from the American cockroach [Protein Data Bank (PDB) ID code 5X0M] (Fig. 7 *C* and *D*). The S2 segment is part of the VSD formed by the first four transmembrane segments, S1–S4; whereas the fifth and sixth transmembrane segments (S5 and S6) are tightly arranged around a fourfold axis of symmetry to create the ion conduction pathway (39–42) (Fig. 7 *A*, *Lower*). VSDs of various voltage-dependent ion channels are endowed with charged amino acids, also called gating charges, and have four highly conserved arginine residues along S4 (e.g., Fig. 7*C*) that mainly contribute to the voltage-driven gating charge transfer during channel activation (43–49). The gating charges reside in aqueous crevices and translocate across a focused electric field that is occluded by a bulky residue (Phe or Tyr) (40–42). It has been suggested that the charge transfer across the Phe/Tyr bulky residue on S2 is facilitated through sequential electrostatic interactions of the gating charge residues with negative countercharges in segments S2 and S3 (50–53). Inspection of the 3D structure indicates that recoding Tyr^189^ into Cys (homologous to the aforementioned Tyr^176^ in PDB ID code 5X0M) would eliminate the bulky occlusion in the pathway of the gating charges in the first VSD (Fig. 7 *C* and *D*), which might modify gating kinetics.

Crz and NPFR neurons also showed differential editing at a site in the voltage-gated potassium channel Shaker (Sh) that is predicted to change Ile^464^ to Val (I464V) in the S6 pore-lining segment. The site showed a 24% increase in editing in Crz neurons and 17% decrease in NPFR neurons compared with the median level of all populations (Fig. 7*E*). Ile^464^ and its flanking amino acids were highly conserved among potassium channels (Fig. 7*F*). To assess the structural effect of such an RNA editing event, we inspected the X-ray crystal structure of the Kv1.2 voltage-dependent K^+^ channel (PDB ID code 2A79) (54), a homologous potassium channel from *Rattus norvegicus*. The 3D structure of the rat Kv1.2 indicated that its Ile^396^ of the S6 pore-lining segment (homologous to Ile^464^ in the *Drosophila* shaker K^+^ channel) forms van der Waals interactions with Leu^331^ and Leu^335^ on the S5 segment of the adjacent subunit (Fig. 7 *G* and *H*). One can therefore envision that the replacement of Ile^396^ by valine in the Kv1.2 structure might result in the loss of the van der Waals interaction with Leu^335^ (Fig. 7 *G* and *H*) and weaken the bond network between S6 and S5. Such a structural perturbation might propagate to the pore helix and the selectivity filter and therefore affect C-type inactivation and/or might propagate along the S6 toward the activation/deactivation gate.

## Discussion

RNA editing is one mechanism that contributes to transcriptomic and proteomic heterogeneity in neurons. We set out to increase the resolution of our understanding of the transcriptome-wide RNA editing landscape within the fly brain by determining editing level differences between nine different neuronal populations. By improving the INTACT protocol, we were able to isolate different populations of neurons with distinct functional differences. Using RNA- and mmPCR-seq, we determined how RNA editing facilitates transcriptomic diversity between these functionally diverse populations.

We identified many previously unknown editing sites in these neuronal populations. These sites, which were often found in lowly expressed transcripts, mostly overlapped repetitive regions of the transcriptome, which is consistent with reports of editing of repeat regions in flies and other species, including Alu sequences in human (55, 56). While most studies of RNA editing in *Drosophila* have focused primarily on ADAR editing of coding regions, our data suggest that ADAR has a wide-ranging role in editing noncoding transcripts. These editing events may regulate transposable elements, circular RNA biogenesis (57), and RNA interference pathways, which can in turn alter heterochromatin formation (55). They may also play a role in the *Drosophila* innate immune system, distinguishing self from nonself RNAs, similar to demonstrated roles for ADAR proteins in mammals (58). While additional studies are needed to determine the functional significance of these editing events, our data suggest that sequencing the transcriptomes of small neuronal populations can facilitate the discovery of these sites by providing deep sequencing of rare RNAs.

We identified hundreds of sites where editing differed between at least two groups of profiled neurons, with Crz and Fru neurons standing out as particularly different from the other populations. In contrast to a report that suggested high editing levels mainly in mushroom body neurons based on editing levels of a reporter construct of an engineered editing substrate (59), we found prominent editing across all of these populations of neurons, with editing levels similar to mushroom body neurons. The editing differences we identified were found at specific sites, rather than being global changes to editing at all sites. These differences could not be explained by *Adar* expression or auto-editing differences between populations or by differential expression of the edited transcripts, suggesting a complex regulation of editing levels at these sites. In some transcripts, we found bidirectional regulation of editing across the same transcript. We also identified a number of editing sites that were physically close and coregulated in the same neuronal populations, as ADAR is likely to edit these groups of sites sequentially. These types of editing differences suggest a regulation of editing that can exert its effect differently in different parts of the same transcript. Regulation by RNA binding proteins may have such an effect (34). We found differential expression of numerous RNA binding proteins between neuronal populations, which may be responsible for some of the differential RNA editing levels we observed at different sites in distinct neuronal populations. Future experiments could determine whether these RNA binding proteins regulate editing levels between cell populations by knocking down these candidate *trans* regulators in populations in which they are differentially expressed and reexamining editing levels in those populations.

In addition to RNA binding proteins, the circadian clock gene *period* has been shown to influence RNA editing in flies (60). Flies with hypomorphic alleles of *Adar* also show defects in circadian rhythm (61), suggesting a connection between RNA editing and circadian rhythm. Interestingly, some Crz neurons have been shown to express *period*, signifying that Crz neurons may play a role in circadian rhythm (62), which could be important for regulation of the editing differences that we observe in Crz neurons. In fact, two related proteins that we found to be differentially edited in Crz neurons, Unc80 and na, are known to play critical roles in circadian rhythm (37). Further functional study is needed to fully determine whether RNA editing in Crz neurons in particular contributes to circadian rhythm.

A number of the differentially regulated sites we identified across these neuronal populations were predicted to be functional in computational analyses of the conservation and natural selection of RNA editing sites across *Drosophila* species (12–14), suggesting that the editing differences we observed between neurons may have physiological consequences for the fly. While evidence of conservation and positive selection are good indications that editing may have functional consequences, further study is needed to understand the physiological effects of these editing events. Based on high homologies with ion channels having resolved 3D structures, we predict that editing of two such sites may alter voltage sensing and gating kinetics in the Paralytic sodium channel and the Shaker potassium channel, presumably leading to functional differences in neuronal excitability or sensitivity to different neuromodulators. The Ile^464^-to-Val editing in the *Drosophila* Shaker K^+^ channel is lowly edited in NPFR neurons and highly edited in Crz neurons. Previous electrophysiological studies showed that, when N-type inactivation is removed, the Val^464^ edited isoform of the *Drosophila* Shaker K^+^ channel displays a significantly slower deactivation rate than the Ile^464^ unedited channel (17). A more physiologically interesting effect emerged when N-type inactivation was characterized in the wild-type edited and unedited isoforms of the Shaker K^+^ channel. That is, compared with the Ile^464^ unedited isoform, the Val^464^ edited isoform inactivates more rapidly, displays stronger steady-state inactivation, and recovers more slowly from inactivation (17). Such alterations in N-type inactivation would likely lead to broadening of the action potentials, as is the case when voltage-gated K^+^ channels in rat mossy fibers inactivate rapidly and recover from inactivation very slowly (63). In fact, editing events nearby, such as the editing site that changes Ile^470^ to Val and is conserved in humans, have profound effects on protein function (22, 64). We therefore hypothesize that the excitability of Crz neurons in the *Drosophila* changes upon Ile^464^ to Val editing in the Shaker K^+^ channel. Crz neurons also show increased editing at Tyr^189^ in the S2 segment of repeat I of the Para channel. Based on 3D modeling, we predict that this change might alter gating kinetics by altering amino acid interactions within the VSD of the protein; however, whether editing would confer different gating properties on the channel remains to be elucidated experimentally.

In addition to protein-recoding differences, we also observed editing changes in 3′ UTRs, which our previous analysis predicts can be functional (12) and might alter gene expression, mRNA localization, or other posttranscriptional regulatory mechanisms. The functional insights provided by our study can prompt future in-depth biochemical and behavioral analyses that were previously hampered by the need to choose which of the thousands of editing sites to focus on. For example, future studies may utilize emerging CRISPR technologies to increase or abolish editing at single sites in particular cell types and then test the effects of the editing changes on subtle phenotypes by using automated behavioral tracking systems such as the Fly Bowl (65). The identification of highly regulated sites and their spatial distribution across different neurons can promote studies to dissect their functional relevance within the right cellular context.

One caveat in considering the functional consequences of these editing sites is taking into account other sites within the same protein that might also alter protein function. We measured editing isoforms for a set of sites that appeared to be coregulated in different neuronal populations, and we found that editing sites that reside within 40 bases of each other in a transcript were often edited in tandem in the same physical transcript. We show here that the Tyr^189^ event in Para is closely linked with another nonsynonymous amino acid change as well as a synonymous change. Since editing events in Shaker have been shown to display functional epistasis (17), this linkage of editing may enhance or attenuate functional consequences of the editing event. We also show that editing of related proteins, such as subunits of voltage-gated calcium channels, can show coregulation within neuronal populations, which might create greater functional differences between these populations.

Decreasing editing at many sites by knocking down ADAR in a number of different neuronal populations leads to locomotor and behavioral changes in the fly (61, 66); however, it is unclear whether the regulation of specific editing sites contribute to these behavioral changes or that it reflects global impairment of neuronal function due to dysregulation of many targets. The data presented in this study serve as a valuable resource toward identifying functionally relevant editing events, as they expose highly regulated sites which can serve to bridge the gap between their cell-specific function and regulation of complex behaviors.

## Methods

### Fly Stocks and Culture.

Flies were raised at 25 °C in a 12-h-light/12-h-dark cycle in 60% relative humidity and maintained on cornmeal, yeast, molasses, and agar medium. *UAS*_*-*_*unc84-2XGFP* transgenic flies were crossed with the following Gal4 drivers: *Dh44-Gal4*, *NPF-Gal4*, *NPFR-Gal4*, *Tdc2-Gal4*, *Crz-Gal4*, *TH-Gal4*, *TRH-Gal4*, *fru-Gal4*, *OK107-Gal4*, and *elav-Gal4*. *NPFR-Gal4* was a gift from the J. Truman laboratory, Howard Hughes Medical Institute Janelia Campus, Ashburn, VA.

### RNA Extractions from Different Neuronal Populations.

Neuronal-population-specific labeled nuclei were isolated by using the INTACT method as described (24) with slight modifications. About 300 adult fly heads (from a mix of males and females) were collected for each sample and homogenized. For Fru samples used as input for mmPCR, male and female heads were collected separately, with sequencing combined later. Nuclear fractions were incubated with anti-GFP antibody and protein G Dynabeads. After washing, bead-bound nuclei were separated by using a magnet and resuspended in Invitrogen Picopure RNA extraction buffer, and RNA was extracted by using the standard protocol. For detailed protocol, see *SI Appendix*, *Extended Methods*.

### mmPCR-Seq and RNA-Seq Library Preparation and Sequencing.

We performed mmPCR-seq as described (25), including a 15-cycle preamplification PCR using 10 μL of cDNA made from INTACT RNA extractions. mmPCR was done using primers designed to amplify *Drosophila* editing sites of interest (33), followed by a 13-cycle barcoding PCR. Samples were pooled, purified, and sequenced on an Illumina NextSeq with paired-end 76-base-pair reads. For RNA-seq, the NuGEN RNAseq v2 kit was used to prepare cDNA from the INTACT purified RNA, followed by library preparation using the SPIA-NuGEN Encore Rapid DR prep kit. Samples were sequenced on an Illumina HiSeq using single-end 60-base-pair reads. Raw sequencing files were deposited in the Gene Expression Omnibus (GEO) database (67) (accession no. GSE113663). See *SI Appendix*, *Extended Methods* for details.

### Identification of Editing Sites.

RNA-seq reads were trimmed and then mapped to the dm6 genome. Mapped reads were processed for indel realignment, duplicate removal, and to call variants. Variants were filtered to remove potential false-positive editing sites. See *SI Appendix*, *Supplementary Note* and *Extended Methods* for details.

### Determining Editing Levels from mmPCR and RNA-Seq.

Sequencing reads were mapped to the dm6 genome before counting the number of A and G base calls from uniquely mapped reads at known and novel editing sites. Editing levels were calculated as (G reads)/(A + G reads) at a site. For Fru neurons, sequencing reads from separately processed male and female heads were combined after we determined there were minimal differences in editing between males and females (*SI Appendix*, Fig. S2). Differences between editing levels were determined by using Fisher’s exact tests comparing A and G counts from one sample to another, with a multiple hypothesis testing correction. “Overall editing levels” were calculated as the number of G reads over the total number of reads at all editing sites for each replicate. We called editing sites population-specific if the absolute values of the z scores for all replicates for one neuronal population were >1.65 and the editing level of that neuronal population was at least 10% different from the next closest population. See *SI Appendix*, *Extended Methods* for details.

### Determining Gene Expression Levels from RNA-Seq.

Reads overlapping exons in each gene were counted, and DESeq2 (68) was used to determine normalized counts and gene expression differences in pairwise comparisons. RNA binding protein expression was clustered based on Pearson’s correlations of the average number of normalized counts between replicates in R. See *SI Appendix*, *Extended Methods* for details.

## Supplementary Material

Supplementary File

Supplementary File

Supplementary File

Supplementary File

Supplementary File

Supplementary File

Supplementary File
