# Supplementary material for: Illuminating spatial A-to-I RNA editing signatures within the Drosophila brain
Source: Proc Natl Acad Sci U S A. 2019 Jan 18;116(6):2318–27. doi: 10.1073/pnas.1811768116 (PMC6369821; doi:10.1073/pnas.1811768116)
Supplement: Supplementary File [file pnas.1811768116.sapp.pdf]

PNAS
www.pnas.org

Supplementary Information for

Illuminating spatial A-to-I RNA editing signatures within the *Drosophila* brain

Anne L. Sapiro, Anat Shmueli, Gilbert Lee Henry, Qin Li, Tali Shalit, Orly Yaron, Yoav Paas, Jin Billy Li, and Galit Shohat-Ophir

Correspondence should be addressed to G.S.O. (galit.ophir@biu.ac.il) or to J.B.L.(jin.billy.li@stanford.edu)

This PDF file includes:

Supplementary text
Figs. S1 to S7
Table S1
Captions for databases S1 to S6
References for SI reference citations

Other supplementary materials for this manuscript include the following:

Datasets S1 to S6

www.pnas.org/cgi/doi/10.1073/pnas.1811768116

Supplementary Information Text

Supplementary Note: Detailed description of de novo editing site discovery

For de novo editing site discovery, we mapped the RNA-seq reads using STAR (1), combined the replicates of each sample for increased coverage, and called variants using GATK (2), filtering out spurious variants as previously reported (3)(**Fig. S1A**). We further required potential sites to be present in at least two of three replicates for each neuronal population. After filtering variants following our previously developed protocols, we found a large number of C-to-T and G-to-A variants and a calculated false discovery rate of 26%. We believed that DNA sequence differences between the parental fly strains of the sequenced F1 progeny were responsible for the large number of variants not caused by A-to-I editing. Because ADAR proteins often edit multiple adenosines in a region (4), particularly in *Drosophila* (5), we required putative editing sites to be adjacent to variants of the same type. This approach was recently shown to aid in distinguishing editing events from SNPs (6). We further filtered variants, requiring two or more of the same type of base conversion within 200 bases of each other, and we found that using this additional filtering step greatly reduced the total number of false positive editing sites called from these datasets with a minimal decrease in sensitivity (**Fig. S1B**). The vast majority (97.7%) of de novo sites filtered out with this step had low coverage across most populations and therefore would not have been considered in our comparative analysis.

After all filtering steps, individual populations had between 85% and 95% A-to-G or T-to-C variants, indicating low numbers of false positive events in all samples. After combining all variants identified in all neuronal populations, 88% were A-to-G or T-to-C. By comparing the number of C-to-T and G-to-A changes to the number of A-to-G and T-to-C changes, we calculated a false discovery rate of 5.6%.

We compared our de novo identified sites to known sites from (7-16). When comparing editing levels between the previously known and newly identified novel sites in the populations in which they were identified, we found that the two groups had similar median editing levels (29% and 26% respectively), but the distribution of editing levels of known sites skewed higher than the novel sites (**Fig. S1C**). The known sites tended to have higher sequencing coverage than the novel sites, as the median coverage of known sites was 105 reads and the median coverage of novel sites was 40 reads (**Fig. S1D**).

Because our de novo editing site discovery pipeline included stringent filters to avoid false positives, we also measured editing levels at previously discovered and characterized editing sites in flies, whether we identified them de novo or not. Doing so allowed us to compare editing at an additional 435 editing sites between neuronal populations. Of those 435 previously known sites, 25 were identified de novo but filtered out because they were not found in clusters. The remaining 410 were filtered out earlier in our pipeline for various other reasons including being close to splice sites, within homopolymeric regions, or overlapping simple repeats, but since they had already been identified and showed reproducible editing levels, we used them in our comparative analysis. To obtain high coverage and accurate editing levels at additional known editing sites that were not covered by RNA-seq, we used mmPCR-seq to amplify sites of interest, and this added coverage of an additional 365 sites that were not identified as high confidence de novo sites.

Extended Methods

RNA extractions from different neuronal populations

Neuronal-population-specific labeled nuclei were isolated using the INTACT method as previously described (17). This method was slightly modified as follows: about 300 adult flies (approximately equal numbers of males and females) were collected from 2-3 day old F1 generation flies of 10 different *Gal4* drivers crossed to *UAS_unc84_2XGFP* and were anesthetized by CO_2 and flash frozen in liquid N_2. For Fru samples used as input for mmPCR, male and female heads were collected separately. Heads were separated by vigorous vertexing followed by separation over dry-ice-cooled sieves. 9ml of homogenization buffer (20mM β-Glycerophosphate pH7, 200mM NaCl, 2mM EDTA, 0.5% NP40 supplemented with RNase inhibitor (SUPERase, Applied Biosystems: AB-AM2696), 10mg/ml t-RNA (ThermoFisher: AM7118), 50mg/ml ultrapure BSA, 0.5mM Spermidine, 0.15mM Spermine) and 140ul of carboxyl Dynabeads M-270 (ThermoFisher: 14305D) was added to each sample. The heads were minced on ice by a series of mechanical grinding steps followed by filtering the homogenate using a 10μm Partek filter assembly (Partek: 0400422314). After removing the carboxyl-coated Dynabeads using a magnet, the homogenate was filtered using a 1μm pluriSelect filter (pluriSelect: 435000103). The liquid phase was carefully placed on a 40% Optiprep (Sigma: D1556) cushion layer and centrifuged in a 4°C centrifuge for 30min at ~2300xg. The homogenate/Optiprep interface

was incubated with anti-GFP antibody (ThermoFisher: G10362) and protein G Dynabeads (ThermoFisher: 100-03D) for 40 minutes at 4^oC. Beads were then washed once in NUN buffer (20mM β-Glycerophosphate pH7, 300mM NaCl, 1M Urea, 0.5% NP40, 2mM EDTA, 0.5mM Spermidine, 0.15mM Spermine, 1mM DTT, 1X Complete protease inhibitor (Sigma: 5056489001), 0.075mg/ml Yeast torula RNA, 0.05Units/μl Superasin (ThermoFisher: AM2696)). Bead-bound nuclei were separated using a magnet stand and resuspended in 100μl of RNA extraction buffer (Picopure kit, ThermoFisher #KIT0204), and RNA was extracted using the standard protocol.

mmPCR-seq and RNA-seq library preparation and sequencing

We performed mmPCR-seq to quantify editing levels at 605 loci harboring known editing sites. We prepared samples for microfluidic PCR with a 15-cycle pre-amplification PCR reaction using 10 μl of cDNA made from INTACT RNA extractions, using the High-Capacity cDNA Reverse Transcriptase Kit (ThermoFisher: 4368814), 6 μl of a pool of all primers used in the multiplex microfluidic PCR, and 4 μl of 5X KAPA2G Fast Multiplex (Kapa Biosystems). The pre-amplification reactions were purified using AMPure XP PCR purification beads (Beckman Coulter). We loaded the pre-amplified samples and 48 pools of PCR primers designed to amplify *Drosophila* editing sites of interest (12), into a 48.48 Access Array IFC (Fluidigm) and performed target amplification as previously described (18). Multiplex PCR products were barcoded using a 13-cycle PCR reaction. After barcoding reaction, samples were pooled and purified using AMPure XP PCR purification beads and were sequenced using Illumina NextSeq with paired-end 76 base-pair reads. For RNA-seq, the NuGEN RNAseq v2 (7102-32) kit was used to prepare cDNA from the INTACT purified RNA, followed by library preparation using the SPIA - NuGEN Encore Rapid DR prep kit (0320-32). Samples were sequenced on an Illumina HiSeq using single-end 60 base-pair reads.

De novo identification of editing sites

To identify novel sites from each neuronal population, we merged RNA-seq reads from three replicates of each neuronal population together as input to our pipeline. We also merged all replicates of all neuronal populations as an additional input to identify novel sites. RNA-seq reads were mapped to the dm6 (Aug 2014, BDGP Release 6 + ISO1 MT/dm6) (19) genome using STAR (v2.4.2) (1) (--twopassMode Basic) after trimming low

quality bases using Trim Galore. Mapped reads were processed using GATK (v3.6) (2) for indel realignment and duplicate removal and to call variants. We removed variants that overlapped known SNPs from the DGRP (20), dbSNP, and a recent study (21), and variants found at the beginning of reads, near splice junctions, in simple repeat regions, or in homopolymeric runs as described in (9). We further filtered variants to remove those with less than 10X coverage, less than 10% editing level, or fewer than 3 alternative nucleotides. We then required variants to be present in at least two of three biological replicates. We removed any variants that were not found next to a variant of the same type in the same transcript (see SI note above). For example, if the nearest variants to an A-to-G change were C-to-T and G-to-A, we would discard the A-to-G, but if one of the adjacent variants was instead an A-to-G both would be kept. All editing sites were annotated using RefSeq gene annotations and ANNOVAR software (22). Editing sites were determined to overlap with repeat regions by comparing site locations to the dm6 RepeatMasker track downloaded from UCSC Table browser (23). To determine protein changes as a result of these editing changes, we used protein annotations from Uniprot (24). RefSeq and Uniprot ID numbers can for highlighted transcripts can be found in **Table S1**.

Determining editing levels from mmPCR-seq and RNA-seq

STAR (v2.4.2) (1) (--twopassMode Basic) was used to map paired-end mmPCR-seq reads and single-end RNA-seq reads to the dm6 genome as described above. We then used the Samtools (25) mpileup function to determine base calls from uniquely mapped reads at known and novel editing sites, and calculated editing levels as number of G reads divided by the total of both A and G reads at a site. For mmPCR-seq, we required each replicate to have 100X coverage and we removed sites that were not within 20% editing between replicates, as done previously (12). Final mmPCR editing levels were determined after downsampling coverage to 200 reads for statistical analysis. For Fru neurons, sequencing reads from separately processed male and female heads were combined after we determined there were minimal differences in editing between males and females (see **Fig. S2**). All other samples, for both mmPCR-seq and RNA-seq, were collected from a mix of male and female heads. For RNA-seq, we required 20X coverage from non-duplicate reads. The majority of sites had either mmPCR-seq or RNA-seq coverage. If we had both mmPCR-seq and RNA-seq coverage at the same editing site, we used the data

from mmPCR-seq only. Differences between editing levels were then determined using Fisher's exact tests comparing A and G counts from one sample to another, with a multiple hypothesis testing correction by p.adjust() using a Benjamini and Hochberg correction (26). Corrected p-values < 0.05 were considered significant. Statistical tests were performed using R (v3.4.1).

For population-specific editing analysis: we called editing sites population-specific if the absolute values of the z-scores for all replicates for one neuronal population were greater than 1.65 and the average editing level of that neuronal population was at least 10% different from the next closest population. Enrichment of nonsynonymous sites and depletion of intronic sites from population-specific editing sites was calculated using GraphPad PRISM 7 Chi-square tests. GO term enrichment was determined using Flymine (27) with a background list of genes set as all genes present in our comparative analysis.

Determining gene expression levels from RNA-seq

Reads overlapping exons in each gene were counted using featureCounts (28), and DESeq2 (29) was used to determine normalized counts and gene expression differences. The DESeq2 function counts(normalized=TRUE) was used to calculate normalized counts with a regularized log transformation. The DESeq() and results() functions were used to calculate gene expression differences between pairs of neuronal populations. RNA binding protein expression was clustered based on Pearson's correlations of the average number of normalized counts between replicates using the R function cor(), and clustered using hclust(method=Ward.D) in R.

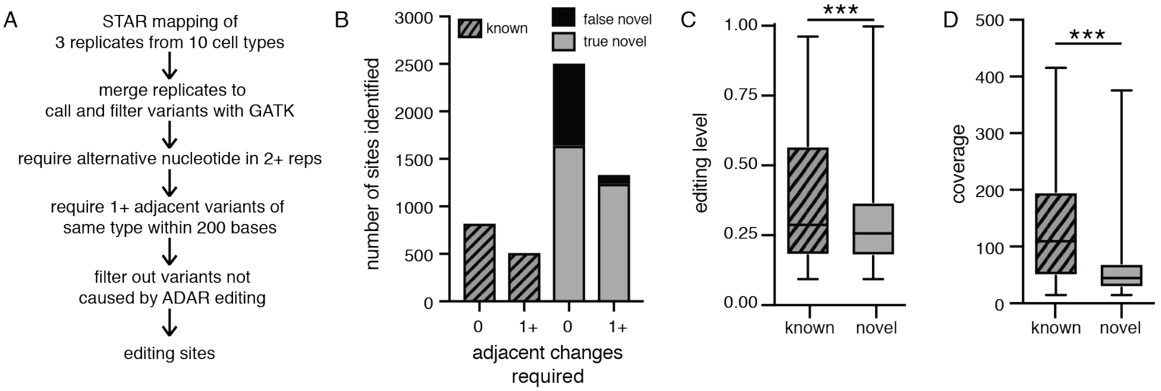

Fig. S1. Pipeline to identify novel sites from RNA-seq. (A) Schematic of analysis pipeline for identifying editing sites from ten neuronal populations. **(B)** The total number of A-to-G or T-to-C variants identified de novo before and after requiring one or more adjacent variants of the same type. On the left are the number of previously known editing sites, on the right are the number of novel sites that are presumed to be true editing sites and the number of novel sites that are presumed false positives based on the calculated false discovery rate. **(C)** Box plots of editing levels at known and novel sites identified de novo in all populations. Whiskers show minimum to maximum values, with boxes representing 25th-75th percentile and median shown. ***$p < 0.0001$, two-tailed Mann-Whitney-U test. **(D)** Box plot of sequencing coverages at known and novel sites identified de novo in all population. ***$p < 0.0001$, two-tailed Mann-Whitney-U test.

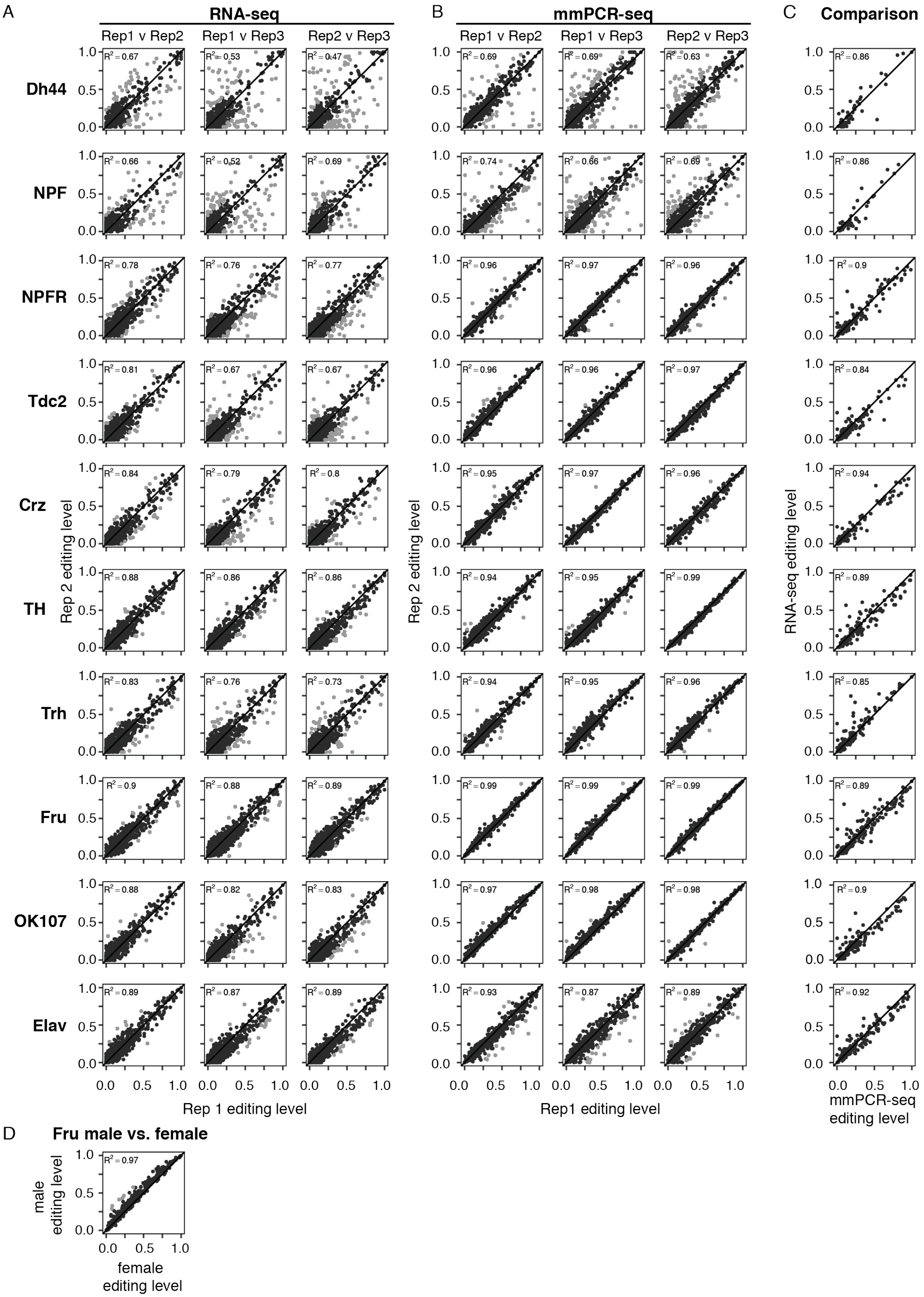
A
RNA-seq
Rep1 v Rep2
Rep1 v Rep3
Rep2 v Rep3
B
mmPCR-seq
Rep1 v Rep2
Rep1 v Rep3
Rep2 v Rep3
C
Comparison
Dh44
NPF
NPFR
Tdc2
Crz
TH
Trh
Fru
OK107
Elav
Dh44: R^2 = 0.67, R^2 = 0.53, R^2 = 0.47, R^2 = 0.69, R^2 = 0.69, R^2 = 0.63, R^2 = 0.86
NPF: R^2 = 0.66, R^2 = 0.52, R^2 = 0.69, R^2 = 0.74, R^2 = 0.66, R^2 = 0.69, R^2 = 0.86
NPFR: R^2 = 0.78, R^2 = 0.76, R^2 = 0.77, R^2 = 0.96, R^2 = 0.97, R^2 = 0.96, R^2 = 0.9
Tdc2: R^2 = 0.81, R^2 = 0.67, R^2 = 0.67, R^2 = 0.96, R^2 = 0.96, R^2 = 0.97, R^2 = 0.84
Crz: R^2 = 0.84, R^2 = 0.79, R^2 = 0.8, R^2 = 0.95, R^2 = 0.97, R^2 = 0.96, R^2 = 0.94
TH: R^2 = 0.88, R^2 = 0.86, R^2 = 0.86, R^2 = 0.94, R^2 = 0.95, R^2 = 0.99, R^2 = 0.89
Trh: R^2 = 0.83, R^2 = 0.76, R^2 = 0.73, R^2 = 0.94, R^2 = 0.95, R^2 = 0.96, R^2 = 0.85
Fru: R^2 = 0.9, R^2 = 0.88, R^2 = 0.89, R^2 = 0.99, R^2 = 0.99, R^2 = 0.99, R^2 = 0.89
OK107: R^2 = 0.88, R^2 = 0.82, R^2 = 0.83, R^2 = 0.97, R^2 = 0.98, R^2 = 0.98, R^2 = 0.9
Elav: R^2 = 0.89, R^2 = 0.87, R^2 = 0.89, R^2 = 0.93, R^2 = 0.87, R^2 = 0.89, R^2 = 0.92
Rep 2 editing level
Rep 1 editing level
Rep1 editing level
RNA-seq editing level
mmPCR-seq editing level
D
Fru male vs. female
R^2 = 0.97
male editing level
female editing level
0.0
0.5
1.0

Fig. S2. Editing levels measured from RNA-seq and mmPCR-seq are reproducible between replicates and consistent with each other. (A-B) Scatter plots of pairwise biological replicates from each of the cell populations from RNA-seq **(A)** and mmPCR-seq **(B)**. Pearson's correlations (R^2) are shown. Sites where editing levels differed by >20% editing between replicates (light gray) were excluded from further analysis because these differences can be caused by technical artifacts, especially from populations with small numbers of neurons like Dh44 and NPF. **(C)** Scatterplot comparisons of the average editing levels between RNA-seq replicates and mmPCR-seq at the subset of sites covered in both. **(D)** Scatterplot comparison of editing levels between Fru male and female heads from mmPCR-seq. Final dataset was created by combining male and female reads.

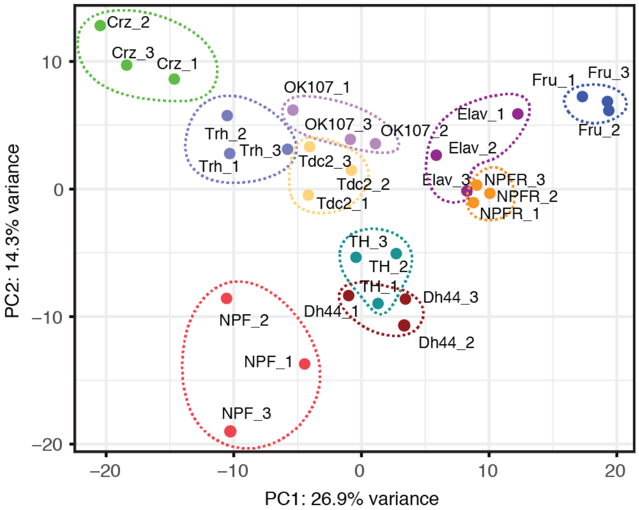

Fig. S3. Principal component analysis of editing levels across populations. Principal component analysis (PCA) of editing levels as measured by either mmPCR-seq or RNA-seq in all replicates at sites that are reproducible between replicates and covered in all samples. PCA was performed using R function prcomp.

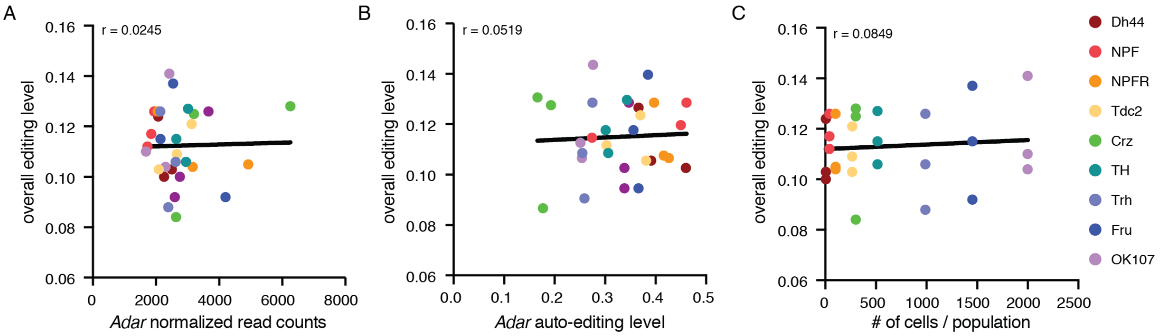

Fig. S4. Overall editing levels do not correlate with *Adar* expression, *Adar* auto-editing, or the number of cells per population. (A) Scatterplot of overall editing level versus to *Adar* normalized read counts for each replicate of RNA-seq. Overall editing levels were calculated as the number of G reads at all editing sites over the total number of A + G reads at all editing sites for each replicate. **(B)** Scatterplot of overall editing level versus *Adar* auto-editing levels for each replicate of RNA-seq. **(C)** Scatterplot of overall editing level versus the number of cells in each neuronal population for each replicate of RNA-seq. Pearson correlations and linear regression lines calculated in GraphPad PRISM 7 are shown.

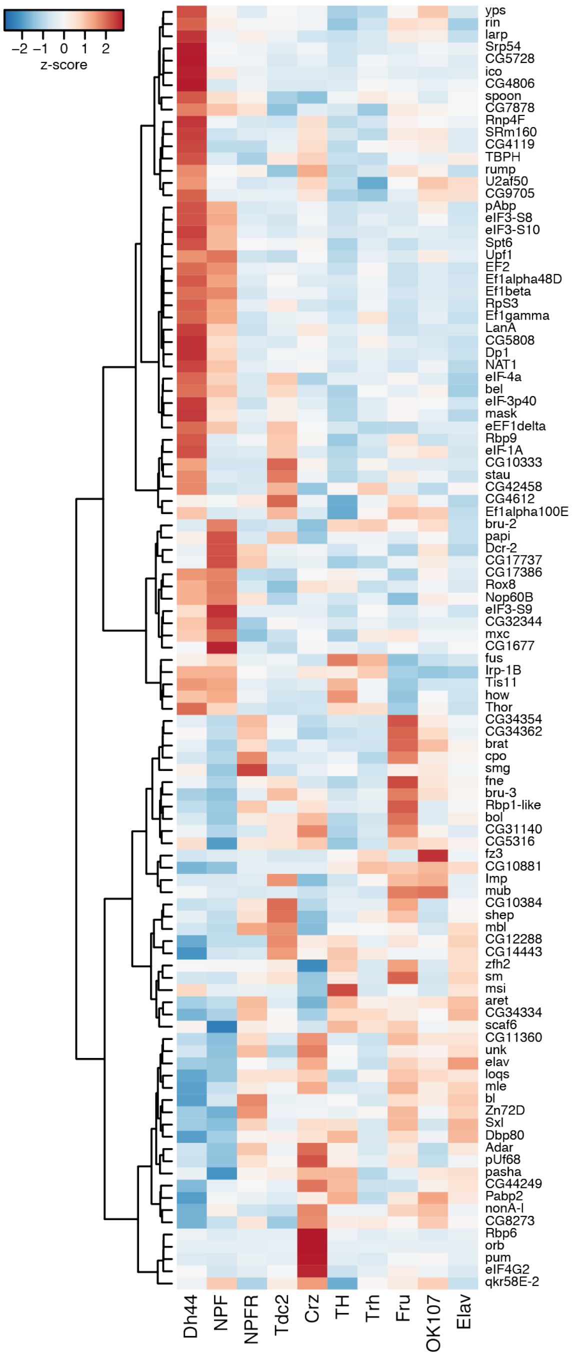

Fig. S5. RNA binding proteins are differentially expressed across neuronal populations. A heat map of gene expression of 105 RNA binding proteins that are differentially expressed by at least twofold (with $p < 0.05$ by Wald tests) between at least two cell populations in pairwise comparisons. Each row represents the expression of the RNA binding protein on the right, as determined by the average normalized number of RNA-seq read counts, across three replicates from each cell population (columns). Z-scores were calculated by normalizing across rows, with blue hues representing less than average expression and red hues representing higher than average expression for each RNA binding protein. The rows were arranged using Ward clustering of Pearson's correlations between expression of RNA binding proteins.

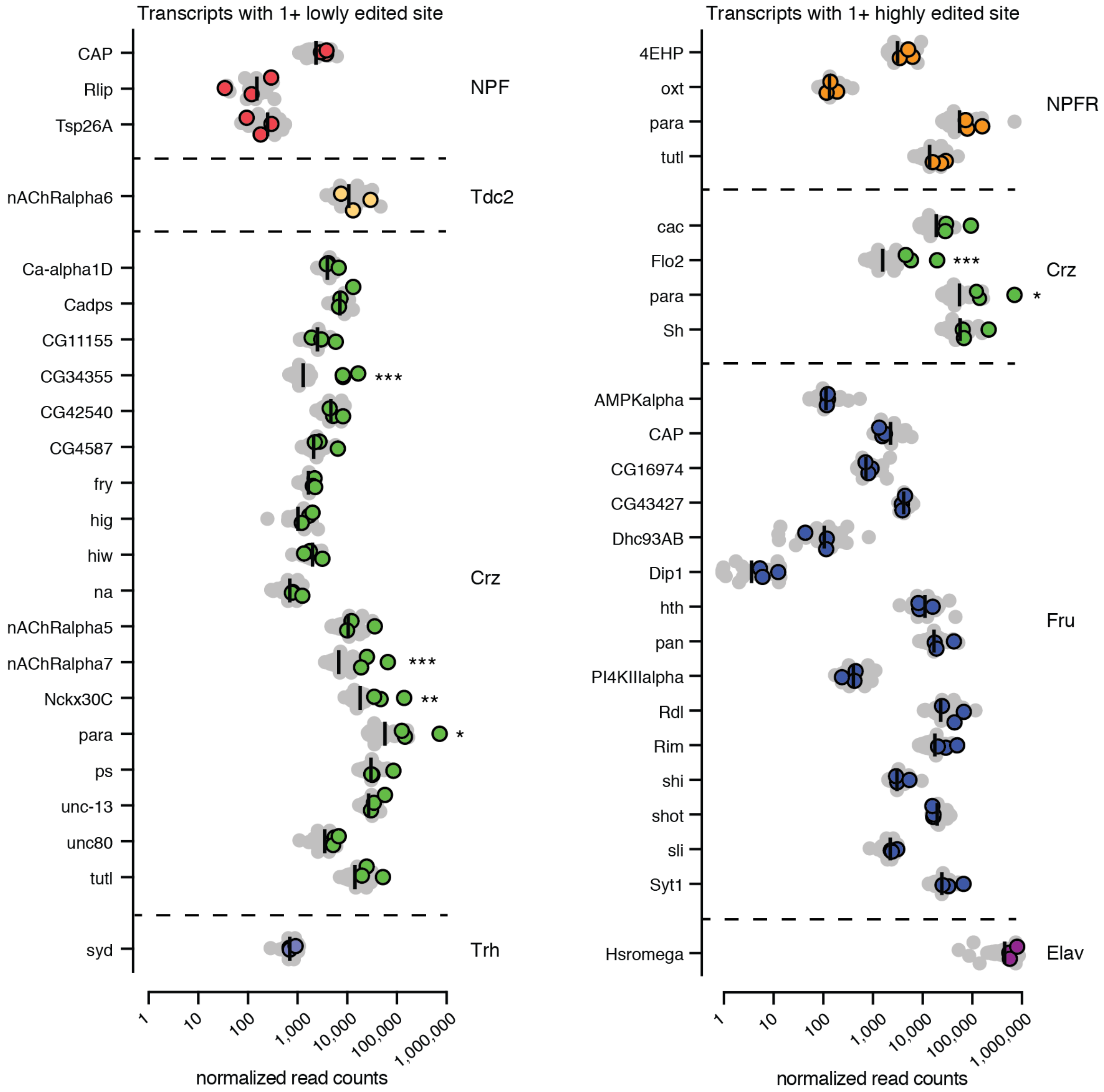

Fig. S6. Expression of transcripts with population-specific editing. Normalized counts of transcripts that have population-specific editing, grouped by the population in which editing is unique (listed on right). Colored dots are three replicates from the population with specific editing, gray are replicates from all other populations, black lines are median counts of all populations. Transcripts with lowly edited sites are on left, and transcripts with highly edited sites on right. X-axis is $\log_{10}$ scale. Significant expression differences were determined through pairwise comparisons between expression in highlighted population versus all other populations using DESeq2. P-values were calculated using Wald tests. *$p < 0.05$ in all pairwise comparisons, **$p < 0.01$ in all pairwise comparisons, ***$p < 0.001$ in all pairwise comparisons.

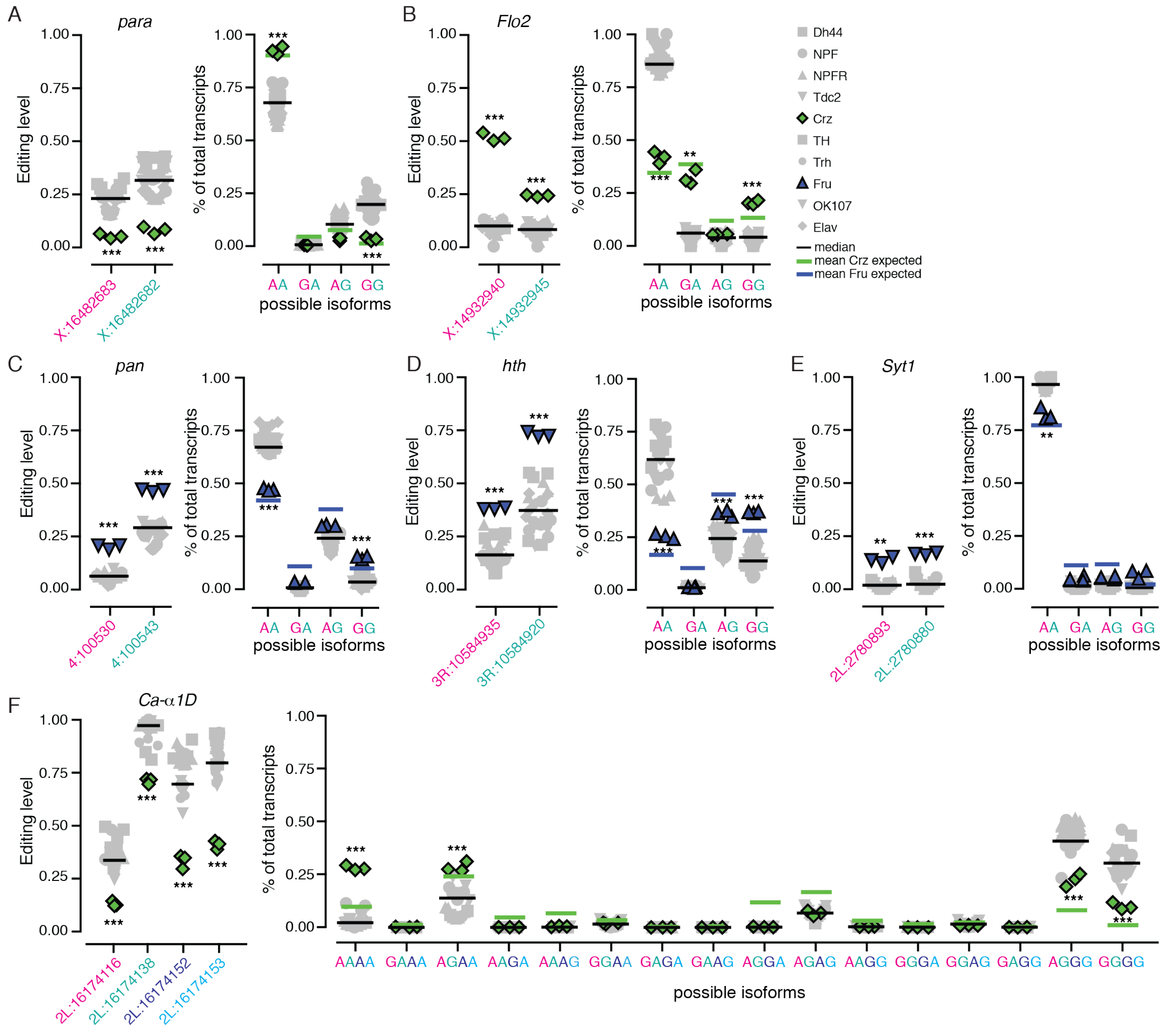

Fig. S7. Coregulation of editing sites. (A-B) Editing levels (left) and isoform usage (right) in clusters of editing sites that are differentially regulated in Crz neurons in *para* and *Flo2*. **(C-E)** Editing levels (left) and isoform usage (right) in clusters of editing sites that are differentially regulated in Fru neurons in *pan*, *hth*, and *Syt1* transcripts. **(F)** Editing levels (left) and isoform usage (right) in cluster of four editing sites that are differentially regulated in Crz neurons in *Ca-alpha1D*. Crz and Fru editing and isoform usage are shown in green and blue respectively, with other populations in gray. Black bars are median editing level of all populations, green and blue bars represent mean expected usage of each isoform in Crz and Fru respectively based on editing levels of the clustered sites. **$p < 0.01$, ***$p < 0.001$ from Welch's t-tests.

Table S1. RefSeq and Uniprot ID numbers for transcripts with editing differences in specific neuronal populations.

Gene Name	RefSeq Transcript ID	Uniprot ID
Ca-alpha1D	NM_134429	Q24270
cacophony	NM_001258710	P91645-1
CG4587	NM_001201888	A8DZ06
na	NM_001103511	A8JUW5
unc80	NM_143320	Q9VB11
nAChRalpha6	NM_205953	Q7KTF8
nAChRalpha7	NM_143320	Q9VWI9
nAChRalpha5	NM_001259098	Q7KT97
paralytic	NM_078647	P35500-1
Shaker	NM_001272736	P08510-1

Dataset S1. Coverage and editing levels of editing sites identified de novo from RNA-seq. (Separate file)

Dataset S2. A and G counts at editing sites and Fisher's exact test p-values from pairwise comparisons between populations. (Separate file)

Dataset S3. Normalized read counts and $\log_2$ fold change calculations and p-values between populations for marker genes, *Adar*, RNA binding proteins, and transcripts with population-specific editing. (Separate file)

Dataset S4. Editing levels, z-scores, and p-values from Welch's t-test for each replicate of all populations. (Separate file)

Dataset S5. GO Term enrichment for transcripts that contain Crz-specific editing events. (Separate file)

Dataset S6. Observed and expected isoform usage of clustered editing sites with p-values from t-tests. (Separate file)

References

1. Dobin A, et al. (2013) STAR: ultrafast universal RNA-seq aligner. *Bioinformatics* 29(1):15–21.

2. McKenna A, et al. (2010) The Genome Analysis Toolkit: a MapReduce framework for analyzing next-generation DNA sequencing data. *Genome Res* 20(9):1297–1303.

3. Ramaswami G, et al. (2012) Accurate identification of human Alu and non-Alu RNA editing sites. *Nat Meth* 9(6):579–581.

4. Levanon EY, et al. (2004) Systematic identification of abundant A-to-I editing sites in the human transcriptome. *Nat Biotechnol* 22(8):1001–1005.

5. Duan Y, et al. (2018) Linkage of A-to-I RNA Editing in Metazoans and the Impact on Genome Evolution. *Mol Biol Evol* 35(1):132–148.

6. Zhang F, Lu Y, Yan S, Xing Q, Tian W (2017) SPRINT: an SNP-free toolkit for identifying RNA editing sites. *Bioinformatics* 33(22):3538–3548.

7. Graveley BR, et al. (2010) The developmental transcriptome of Drosophila melanogaster. *Nature* 471(7339):473–479.

8. Rodriguez J, Menet JS, Rosbash M (2012) Nascent-seq indicates widespread cotranscriptional RNA editing in Drosophila. *Molecular Cell* 47(1):27–37.

9. Ramaswami G, et al. (2013) Identifying RNA editing sites using RNA sequencing data alone. *Nat Meth* 10(2):128–132.

10. St Laurent G, et al. (2013) Genome-wide analysis of A-to-I RNA editing by single-molecule sequencing in Drosophila. *Nat Struct Mol Biol* 20(11):1333–1339.

11. Sapiro AL, Deng P, Zhang R, Li JB (2015) Cis regulatory effects on A-to-I RNA editing in related Drosophila species. *Cell Reports* 11(5):697–703.

12. Ramaswami G, et al. (2015) Genetic mapping uncovers cis-regulatory landscape of RNA editing. *Nat Commun* 6:8194.

13. Mazloomian A, Meyer IM (2015) Genome-wide Identification and Characterisation of Tissue-specific RNA Editing Events in D. melanogaster and their Potential Role in Regulating Alternative Splicing. *RNA Biol* 12(12): 1391–1401.

14. Zhang R, Deng P, Jacobson D, Li JB (2017) Evolutionary analysis reveals regulatory and functional landscape of coding and non-coding RNA editing. *PLoS Genet* 13(2):e1006563.

15. Duan Y, Dou S, Luo S, Zhang H, Lu J (2017) Adaptation of A-to-I RNA editing in Drosophila. *PLoS Genet* 13(3):e1006648.

16. Yu Y, et al. (2016) The Landscape of A-to-I RNA Editome Is Shaped by Both Positive and Purifying Selection. *PLoS Genet* 12(7):e1006191.

17. Henry GL, Davis FP, Picard S, Eddy SR (2012) Cell type-specific genomics of Drosophila neurons. *Nucleic Acids Research* 40(19):9691–9704.

18. Zhang R, et al. (2014) Quantifying RNA allelic ratios by microfluidic multiplex PCR and sequencing. *Nat Meth* 11(1):51–54.

19. Hoskins RA, et al. (2015) The Release 6 reference sequence of the Drosophila melanogaster genome. *Genome Res* 25(3):445–458.

20. Huang W, et al. (2014) Natural variation in genome architecture among 205 Drosophila melanogaster Genetic Reference Panel lines. *Genome Res* 24(7):1193–1208.

21. Yablonovitch AL, et al. (2017) Regulation of gene expression and RNA editing in Drosophila adapting to divergent microclimates. *Nat Commun* 8(1):1570.

22. Wang K, Li M, Hakonarson H (2010) ANNOVAR: functional annotation of genetic variants from high-throughput sequencing data. *Nucleic Acids Research* 38(16):e164.

23. Karolchik D, et al. (2004) The UCSC Table Browser data retrieval tool. *Nucleic Acids Research* 32(Database issue):D493–496.

24. The UniProt Consortium (2018) UniProt: the universal protein knowledgebase. *Nucleic Acids Research*. 46(5):2699.

25. Li H, et al. (2009) The Sequence Alignment/Map format and SAMtools. *Bioinformatics* 25(16):2078–2079.

26. Benjamini Y, Hochberg Y (1995) Controlling the False Discovery Rate: A Practical and Powerful Approach to Multiple Testing. *Journal of the Royal Statistical Society Series B (Methodological)* 57(1):289–300.

27. Lyne R, et al. (2007) FlyMine: an integrated database for Drosophila and Anopheles genomics. *Genome biology* 8(7):R129.

28. Liao Y, Smyth GK, Shi W (2014) featureCounts: an efficient general purpose program for assigning sequence reads to genomic features. *Bioinformatics* 30(7):923–930.

29. Love MI, Huber W, Anders S (2014) Moderated estimation of fold change and dispersion for RNA-seq data with DESeq2. *Genome biology* 15(12):550.
